# Different metabolite profiles across *Penicillium roqueforti* populations associated with ecological niche specialisation and domestication

**DOI:** 10.1186/s43008-024-00167-4

**Published:** 2024-11-28

**Authors:** E. Crequer, E. Coton, G. Cueff, J. V. Cristiansen, J. C. Frisvad, R. C. Rodríguez de la Vega, T. Giraud, J.-L. Jany, M. Coton

**Affiliations:** 1 Laboratoire Universitaire de Biodiversité Et Ecologie Microbienne, Univ. Brest, INRAE, 29280 Plouzane, France; 2 Department of Biotechnology and Biomedicine, Technical University of Denmark, 2800 Kongens Lyngby, Denmark; 3 Laboratoire Ecologie Systématique et Evolution, UMR 8079, AgroParisTech, Université Paris-Saclay, CNRS, Bâtiment 680, 12 Route RD 128, 91190 Gif-Sur-Yvette, France

**Keywords:** Fungal metabolites, Metabolomics, Secondary metabolites, Mycotoxins, Domestication, Adaptation, Food safety

## Abstract

Fungi are known to produce many chemically diversified metabolites, yet their ecological roles are not always fully understood. The blue cheese fungus *Penicillium roqueforti* thrives in different ecological niches and is known to produce a wide range of metabolites, including mycotoxins. Three *P. roqueforti* populations have been domesticated for cheese production and two populations thrive in other anthropized environments, i.e., food, lumber and silage. In this study, we looked for differences in targeted and untargeted metabolite production profiles between populations using HPLC-HR-Q-TOF and UHPLC-Q-TOF-HR-MS/MS. The non-cheese populations produced several fatty acids and different terpenoids, lacking in cheese strains. The Termignon cheese population displayed intermediate metabolite profiles between cheese and non-cheese populations, as previously shown for other traits. The non-Roquefort cheese population with the strongest domestication syndrome, produced the lowest quantities of measured metabolites, including mycophenolic acid (MPA), andrastin A and PR toxin. Its inability to produce MPA was due to a deletion in the *mpaC* gene, while a premature stop codon in ORF 11 of the PR toxin gene cluster explained PR toxin absence and the accumulation of its intermediates, i.e., eremofortins A and B. In the Roquefort population, we detected no PR toxin nor eremofortins A or B, but found no indel or frameshift mutation, suggesting downregulation. The hypotoxigenic trait of domesticated cheese populations can be hypothesized to be linked to the loss of this ability through trait degeneration and/or the selection of low toxin producers. It may also be due to the fact that populations from other anthropized environments maintained high metabolite diversity as the bioactivities of these compounds are likely important in these ecological niches.

**Supplementary Information:**

The online version contains supplementary material available at 10.1186/s43008-024-00167-4.

## Introduction

Fungi are known to produce a wide range of chemically diversified metabolites that are crucial for their development, interactions, survival and/or competition with other microorganisms in complex ecosystems. These metabolites include toxins, antimicrobial compounds, and molecules involved in communication or protection from UV damage (Keller 2019; Stroe et al. 2023). Some of these metabolites also exhibit significant activities outside their ecological role, such as antibiotics, anti-cancer agents and immunosuppressants (Keller 2019; Stroe et al. 2023). However, the role of these metabolites is still not fully understood and identifying different metabolite profiles in populations thriving in distinct niches may contribute to our understanding of their ecological role.

*Penicillium roqueforti* is a highly interesting filamentous fungus from an ecological point of view, as it colonises a multitude of niches, with different populations presenting adaptive differentiation (Gillot et al. 2015; Dumas et al. 2020; Crequer et al. 2023). This species is well known worldwide for its role in blue cheese production (Gillot et al. 2015, 2017b; Dumas et al. 2020). However, *P. roqueforti* has also been isolated from lumber and is a common contaminant in silage, as well as various food products such as dairy, fruits and baked food (Pitt and Hocking 2009; Crequer et al. 2023). *Penicillium roqueforti* produces a wide variety of chemically diverse metabolites, including many so-called secondary metabolites, now also referred to as specialised metabolites, which have known bioactive properties (Pichersky et al. 2006; Pichersky & Lewinsohn 2011). Some of these metabolites correspond to mycotoxins, the most toxic one being the aristolochene-derived sesquiterpene PR toxin (*P. roqueforti* toxin), which can be a threat to animal feeding and human food safety. For example, the PR toxin in silage causes liver toxicity or subacute symptoms in livestock (Gallo et al. 2015; Hymery et al. 2017; Dubey et al. 2018). In cheese, the PR toxin is considered unstable (Scott & Kennedy 1976) and apparently degraded to PR imine (Siemens and Zawistowski 1993), a molecule with lower toxicity (Hymery et al. 2014). *Penicillium roqueforti* also produces the alkaloid mycotoxin roquefortine C (ROQ C) and the meroterpenoid mycophenolic acid (MPA), which can be found in various cheeses in a wide range of concentrations (Scott and Kennedy 1976; Lafont et al. 1979; Engel et al. 1982; Finoli et al. 2001; Kokkonen et al. 2005; Usleber et al. 2008; Fontaine et al. 2015). These compounds have relatively low cytotoxic effects compared to mycotoxins regulated in food by the European Union, such as aflatoxins, ochratoxin A or patulin (Commission regulation European Union N° 2023/905) (Fontaine et al. 2016). Mycophenolic acid is even widely used in the medical field as a treatment to prevent organ transplant rejection. Andrastin A (AND A) is another meroterpenoid from *P. roqueforti* with promising anticancer activity (Nielsen et al. 2005). Additional secondary metabolites, such as clavines, terpenoids, alkaloids and peptides, may also play a role in *P. roqueforti* fitness in different environments. For example, two tetrapeptides, Phe-Val-Val-Phe and Phe-Val-Val-Tyr, have antimicrobial properties likely important for interspecies competition (Hammerl et al. 2019). Studying the differences in metabolite production between *P. roqueforti* populations may help gain a more general understanding of their ecological role in diverse niches.

Five populations of *P. roqueforti* have been identified, corresponding to three cheese populations and two non-cheese populations (Dumas et al. 2020; Crequer et al. 2023). A first cheese population, called non-Roquefort, corresponds to a clonal lineage (Dumas et al. 2020), and is used worldwide for the production of most kinds of blue cheeses (*e.g.* Gorgonzola, Cabrales, Stilton and Danablue). This population presents numerous beneficial traits for large-scale cheese making, such as higher salt and lactic acid tolerance, faster growth on cheese and faster lipolysis (Dumas et al. 2020; Caron et al. 2021; Crequer et al. 2023). A second cheese population is mainly associated with the Roquefort protected designation of origin (PDO) (Gillot et al. 2015; Dumas et al. 2020). This Roquefort population harbours slightly higher genetic diversity and displays traits beneficial for cheese making following more traditional processes, such as longer conservation and growth on bread (Dumas et al. 2020). More recently, a third cheese population was identified in Termignon blue cheeses which are not inoculated with *P. roqueforti* spores but instead spontaneously colonise these specific cheeses from the environment in the French Alps. Termignon strains exhibit intermediary phenotypic traits between the cheese and non-cheese populations and likely correspond to descendants of an ancient population with mild domestication syndrome (Crequer et al. 2023). Two genetically different non-cheese populations have been identified, one mostly associated with silage, as well as to a lesser extent spoiled food, and the other with spoiled food and lumber (Dumas et al. 2020). These populations exhibit much higher genetic diversity than the cheese populations and have differentiated from each other more recently than from the cheese populations (Dumas et al. 2020).

These genetically differentiated populations of *P. roqueforti* thriving in contrasting environments constitute a great model for studying adaptation to different substrates, particularly the potential ecological roles of the metabolites they produce in cheese versus other anthropized environments. The non-Roquefort and Roquefort cheese populations result from two distinct domestication events (Dumas et al. 2020), in the contrasting contexts of more industrial and more traditional production processes, respectively. As a result of diverging selection, metabolite production and their underlying genetic mechanisms, may have differed. In this study, we therefore compared the metabolite production profiles between the five *P. roqueforti* populations using both targeted metabolomics for seven known metabolites, including mycotoxins, and untargeted metabolomics. We also explored the genetic mechanisms underlying the differences using available genomic data.

## Materials and methods

### Strain collection and conidium suspension preparation

For metabolite profiling, we randomly chose 44 strains from the five known *P. roqueforti* populations (Dumas et al. 2020; Crequer et al. 2023): twelve strains from the non-Roquefort population, eight from the Roquefort population and ten from each of the lumber/spoiled food and the silage/spoiled food populations. We also used the four available strains sampled from Termignon blue cheeses. All strains are available in the ESE (Ecology Systematics and Evolution, Paris Saclay university) or UBOCC (https://nouveau.univ-brest.fr/ubocc/fr) culture collections (Additional file 1: Table S1).

Conidium suspensions were prepared for the various experiments by cultivating the fungal strains for six days at 25 °C on potato dextrose agar (PDA, Difco, Fisher Scientific). Two mL of Tween 80 (0.045%, v/v) were then added on each plate and conidia were scraped off the surface. Conidium concentrations in the suspensions were estimated using Malassez cells and adjusted to 5.10^5^ conidia.mL^−1^ with Tween 80, in 20% glycerol. Suspensions were then stored at −80 °C for cultures for metabolite extraction.

### Metabolite extraction

For metabolite production measurements, we grew fungal cultures in 24-well sterile microplates containing two mL of yeast extract sucrose (YES) agar medium buffered at pH 4.5 with phosphate-citrate buffer and characterised by a high C/N ratio that increases metabolite production in *Penicillium* fungi (Frisvad and Filtenborg 1983). For each strain, 2 µL of the previously prepared spore suspension was inoculated in the centre of the well. Six replicates per strain were performed: three for secondary metabolite analyses and three for fungal dry-weight measurements. For fungal dry-weights, growth was performed on cellophane disks to collect fungal mycelium. The plates were incubated at 25 °C in the dark for ten days and then stored at −20 °C until dry-weight measurement or metabolite profiling. For metabolite extractions, we used an optimised high-throughput extraction method (Gillot et al. 2017b; Lo et al. 2023). After thawing, we homogenised 2 g aliquots (the entire YES culture with mould obtained from a well) with a sterile flat spatula, to which we added 12.5 mL of acetonitrile (ACN) supplemented with 0.1% formic acid (v/v); samples were agitated at 175 rpm and 25 °C for 90 min followed by 15 min sonication. The extracts were vortexed again before centrifugation for 10 min at 5000 g at 4 °C. The supernatants were then collected and filtered through 0.45 µm polytetrafluoroethylene membrane filters into amber vials and stored at −20 °C until analysis.

### Targeted secondary metabolite detection and quantification

Targeted secondary metabolite characteristics used for quantifications are given in Table S3 and included commercially available standards produced by *Penicillium* species: andrastin A (AND A), eremofortins A and B (ERE A & B), (iso)-fumigaclavine A (FUM A), mycophenolic acid (MPA) and roquefortin C (ROQ C). AND A, ERE A & B and FUM A standards were obtained from Bioviotica (Goettingen, Germany), and others from Sigma-Aldrich (St Louis, MO, USA). All stock solutions were prepared in dimethyl sulfoxide (DMSO) at 1 mg.mL^−1^ in amber vials. For these analyses, metabolite identification was performed using both the mean retention time ± 1 min and the corresponding ions listed in Table S2 (additional file 2). We used a matrix-matched calibration curve for reliable secondary metabolite quantification with final concentrations ranging from 1 to 10000 ng.mL^−1^ according to the target metabolite and method performance as previously described (Gillot et al. 2017b). All metabolite concentrations were determined using the Agilent MassHunter Workstation Software (Agilent Technologies, Sanat Clara, CA, USA) with a linear regression model. Specific mycotoxin production was expressed as ng per g of extracted matrix and mg of fungal dry weight (ng.g^−1^.mg^−1^). For PR toxin, a purified solution with unknown concentration was previously obtained (Gillot et al. 2017b) and diluted 1X, 2X, and 5X (taking into account matrix effect) to ensure peak separation and determine the detection limit. According to the international council for harmonisation guidelines (ICH Harmonised Tripartite Guideline 2005), detection and quantification limits of each metabolite were obtained by multiplying the standard deviation of y intercepts of regression lines divided by the slope, by 3.3 and 10, respectively. Means per strain across replicates (Additional file 3 and 4: Tables S3 and S4) were used to compare populations. Electrospray ionization (+/-) modes were both systematically performed on all samples. Method performance characteristics were obtained for the seven targeted metabolites prepared in blank extraction of YES medium and all seven metabolites were eluted at different retention times, including MPA and PR toxin known to have a quantifying ion with the same mass (m/z 321.13; additional file 2: Table S2). Linearity (R^2^) for each standard curve was determined to be above 0.982 (additional file 2: Table S2) for all metabolites in electrospray ionisation positive mode, ESI +, with better performance for detection in ESI + than ESI -.

Targeted analyses were performed on an Agilent 6530 Accurate-Mass Quadrupole Time-of-Flight mass spectrometry system equipped with a binary pump 1260 and degasser (Q-TOF LC/MS), well plate autosampler set to 10 °C and a thermostatted column compartment. Filtered 2 µL aliquots were injected into a ZORBAX Extend C-18 column (2.1 × 50 mm and 1.8 µm, 600 bar) maintained at 35 °C with a flow rate set to 0.3 mL.min^−1^. The mobile phase A contained milli-Q water + 0.1% formic acid (v/v) and 0.1% ammonium formate (v/v) while mobile phase B was ACN + 0.1% formic acid. Mobile phase B was maintained at 10% for 4 min followed by a gradient from 10 to 100% for 36 min. Then, mobile phase B was maintained at 100% for 5 min before a 5 min post-time. Samples were ionised in both positive (ESI+) and negative (ESI-) electrospray ionisation modes in the mass spectrometer with the following parameters: capillary voltage 4 kV, source temperature 325 °C, nebulizer pressure 50 psig, drying gas 12 L.min^−1^, ion range 100–1000 m/z.

### Untargeted metabolite analysis, data processing and metabolite identifications UHPLC-Q-TOF-HRMS/MS analysis

We also used an untargeted metabolomics approach with an ultra-high-performance liquid chromatography-diode array detection - quadrupole time of flight mass spectrometry (UHPLC-DAD-Q-TOF-MS/MS) during metabolite profiling on three *P. roqueforti* extracts, the lumber L6, Roquefort R3 and Termignon T4 strains (biological triplicates). These extracts were selected based on the above mentioned targeted LC-QTOF analyses as they also displayed multiple unknown compounds and the three extracts covered the full spectrum of these observed unknown metabolites.

We detected metabolites using an Agilent 6545 Quadrupole Time-of-Flight (Q-TOF) MS equipped with an UHPLC Agilent Infinity 1290 (Agilent Technologies, Santa Clara, CA, 502 USA) including a diode array detector. Separation was done on a Poroshell 120 Phenyl Hexyl column (150 × 2.1 mm i.d., 1.9 μm; Agilent Technologies, Santa Clara, CA) maintained at 40°C. Samples injected (1 μL) were eluted with a flow rate set to 0.35 mL.min^−1^ using a linear gradient increasing from 10% acetonitrile (LC-MS grade) in Milli-Q water supplemented with 20 mM formic acid to 100% over the first 10 min, maintaining 100% for 2 min before decreasing back to 10% in 0.1 min and holding initial conditions for 3 min before the next run. The Agilent accurate-mass 6530 Quadrupole Time-of-Flight (Q-TOF) liquid chromatography/mass spectrometer (LC/MS) system was equipped with an Agilent Dual Jet Stream electrospray ion source (ESI) with a drying gas temperature set to 250°C and flow of 12 L.min^-1^. Samples were ionised in positive (ESI+) electrospray ionisation modes in the mass spectrometer with the following parameters: capillary voltage 4 kV, nozzle voltage 500 V, ion range 100–1000 m/z and auto MS/MS fragmentation at three collision energies (10, 20 and 40 eV). The acquisition rate was set to 10 spectra per second and MS spectra were recorded as centroid data. Reference masses (two [M+H]+ ions were: 186.2216 and 922.0098) were injected in the second sprayer using a supplementary LC pump at 15 µl.min-1 flow rate using a 1:100 splitter.

### LC-MS/MS data processing

The generated mass spectrometry (MS) data, recorded as centroid data, were analysed using both an in-house library search with the Agilent MassHunter PCDL manager and MZmine3, GNPS (global natural product social molecular networking*)* and SIRIUS tools available on GitHub (https://github.com). For MZmine3 analyses, centroid data were converted into the community standard for mass spectrometry data, mzML, using the ProteoWizard software (version 3.0.22112, MSConvert tool) (Martens et al. 2011; Chambers et al. 2012). MZmine3 software (Schmid et al. 2023) was then used to process mzML files and the batch file that was created included the different processing steps as feature detection, deconvolution and filtering. The final feature quantification table, exported in the MGF (mascot generic format) standard format, was used in the GNPS Networking web-based mass spectrometry ecosystem (https://gnps.ucsd.edu/) to generate a molecular network for feature determinations using Cytoscape v3.9.1 (https://cytoscape.org/) and SIRIUS 5.6.3 (https://bio.informatik.uni-jena.de/software/sirius/; (Dührkop et al. 2019)) softwares for further downstream analyses and chemical family predictions and/or identifications.

After data processing of UHPLC-Q-TOF-MS/MS spectra, the LC-Q-TOF/MS spectra for the same three strains were also analysed using SIRIUS 5.6.3 (Dührkop et al. 2019) and the two data sets were compared to match both data sets together (*i.e.* identify common ions), determine putative formulas and compare MS-MS spectra against databases available in SIRIUS to predict the metabolite family and, when possible, identify metabolites. This comparison allowed us to then transpose this data to all the generated metabolite data and extract identified ion peak areas from all LC-Q-TOF spectra. We were therefore able to obtain the metabolite production profile per strain, which was used to compare metabolite production profiles between populations. Control samples were also included, corresponding to the blank YES medium (no mould); it was used to extract and distinguish compounds produced by strains from compounds originally present in the medium. The compounds extract area was than normalized with the corresponding strain mycelium mass before statistical analyses (expressed in area.mg^-1^ of mycelium; additional file 4: Table. S4).

### Statistical analyses

Statistical analyses for testing differences in metabolite production and dry weight between populations were performed using the R software (version 4.2.1, https://www.r-project.org/). Shapiro-Wilk and Bartlett tests (package *rstatix*, R) were performed to assess normality and homoscedasticity of residuals in each population. If the data, the racine-transformed data or the log-transformed data did not deviate from normality, populations were compared using ANOVA type I and Tukey tests were used as *post-hoc* tests. If the data, the log-transformed data and square-root transformed data significantly deviated from normality, a Kruskall-Wallis test was performed on raw data to compare populations, followed by Dunn tests as *post-hoc* tests (additional file: Table. S5).

### Comparative analysis of secondary metabolite biosynthetic gene clusters

In order to study the targeted metabolite biosynthetic gene clusters, we used the annotations of the LCP06136 (Caron et al. 2024) and FM164 reference genomes (genbank accession number GCA_000513255.1), and we used the genomes of all other strains analysed here that were previous assembled from Illumina data (Dumas et al. 2020; Crequer et al. 2023). We lifted the annotations of the known gene clusters controlling the production of the studied metabolites, MPA, PR toxin, FUM A, AND A and ROQ C, from the two reference genomes to the Illumina genomes (accession numbers in Table S1), for all the phenotyped strains, using *liftoff* v1.6.3 (Shumate & Salzberg 2021). Protein sequences and coding DNA sequences (CDS) of each gene were extracted from each genome using *gffread* v0.12.1 (Pertea and Pertea 2020). Reference gene annotation used corresponded to the annotation with the longest CDS, to be conservative regarding the likelihood to detect complete genes. Protein and CDS sequences were aligned using *mafft* v7.475 (Katoh and Standley 2013) and analysed using *Jalview* 2.11.2.0 (Waterhouse et al. 2009; Troshin et al. 2018).

## Results

### Metabolite profiles are different between P. roqueforti populations

We performed targeted and untargeted LC-Q-TOF metabolite profiling on 44 *P. roqueforti* strains from the five identified *P. roqueforti* populations (Table S1), and identified metabolites of interest using in-house local databases or *in silico* global natural products social molecular networking (GNPS) molecular networks and SIRIUS classification. Based on local databases, we identified 16 metabolites (Table 1): agroclavine, AND A, FUM A, MPA-associated molecules (MPA, MPA isomer, homo MPA, MPA prenyl and MPA IV), roquefortines C and D, PR toxin, eremofortins A and B, and three tetrapetides, i.e., cyclo(Phe-Val-Val-Phe), Phe-Val-Val-Phe and Phe-Val-Val-Tyr. We also predicted the chemical classes for 20 other metabolites, including eight fatty acids, among which some fatty acid amides, 11 terpenoids, among which a putative eremofortin C, and one alkaloid (putative festuclavine), while 11 others remained as unidentified metabolites (Table 1). Among the fatty amides detected, two were recently described as potential contaminants from plastic tubes. These fatty amides are N‐lauryldiethanolamine and N‐(2‐hydroxyethyl)‐N‐(2‐(2‐hydroxyethoxy)ethyl)dodecylamine, referred to as “putative lauryldiethanolamine” and “fatty acid 7”, respectively, in our study. Following the findings of Chai et al. (2019), we decided to exclude these compounds from further analyses to avoid potential contamination effects, despite their absence in the control (YES medium blank extract).

**Table 1:** Untargeted metabolite characteristics: Retention times and masses in quantification runs in HPLC-QTOF and identification runs with UHPLC-QTOF, Sirius prediction (in silico MS/MS match) and identification against local databases specific to fungus metabolites (MS/MS match)

Name	LC-Q-TOF		UHPLC-Q-TOF		Sirius prediction				Name	Name
	Retention time	Mass	Retention time	Mass	Molecular formula	adduct	NPC#pathway	NPC #pathway Probability		
Putative agroclavine	3.172	239.152	3.585	239.154	C16H18N2	[M + H] +	Alkaloids	0.998	Agroclavine	MS/MS match
Putative festuclavine	4.325	241.172	3.778	241.171	C16H20N2	[M + H] +	Alkaloids	0.998	–	–
cyclo(PheValValPhe)	23.124	493.276	6.922	493.280	C28H36N4O4	[M + H] +	Amino acids and Peptides	0.986	cyclo-(Phe-Val-Val-Phe	MS/MS match
D-Phe-L-Val-D-Val-L-Phe	16.652	511.287	5.028	511.291	C28H38N4O5	[M + H] +	Amino acids and Peptides	0.999	tetrapeptide-(Phe-Val-Val-Phe)	MS/MS match
D-Phe-L-Val-D-Val-L-Tyr	13.707	527.281	4.211	527.286	C28H38N4O6	[M + H] +	Amino acids and Peptides	0.999	tetrapeptide-(Phe-Val-Val-Tyr)	MS/MS match
MPA isomer	24.548	375.213	7.184	375.216	C22H30O5	[M + H] +	Polyketides	0.748	Mycophenolic acid isomer	MS/MS match
homoMPA	22.050	385.159	6.597	385.162	C20H26O6	[M + Na] +	Polyketides	0.769	Homomycophenolic acid	MS/MS match
MPA prenyl	32.294	385.234	8.671	385.238	C24H32O4	[M + H] +	Polyketides	0.745	6-Farnesyl-5,7-dihydroxy-4-methylphthalide; MPA prenyl	MS/MS match
MPA IV	23.980	389.192	7.034	389.196	C22H28O6	[M + H] +	Terpenoids	0.736	Mycophenolic acid IV (version II)	MS/MS match
Roquefortin D	11.017	392.204	4.072	392.207	C22H25N5O2	[M + H] +	Alkaloids	0.999	Roquefortine D	MS/MS match
Putative lauryldiethanolamine	19.403	274.271	5.952	274.274	C16H35NO2	[M + H] +	Fatty acids	0.958	–	in silico MS/MS match
Fatty acid 1	19.526	230.246	5.950	230.248	C14H31NO	[M + H] +	Fatty acids	0.978	–	in silico MS/MS match
Fatty acid 2	0.538	232.152	1.802	232.154	C11H21NO4	[M + H] +	Fatty acids	0.999	–	in silico MS/MS match
Fatty acid 3	25.063	279.229	7.034	279.232	C18H30O2	[M + H] +	Fatty acids	0.971	–	in silico MS/MS match
Fatty acid 5	19.800	290.267	6.026	290.269	C16H35NO3	[M + H] +	Fatty acids	0.978	–	in silico MS/MS match
Fatty acid 6	18.773	295.224	6.484	295.226	C18H30O3	[M + H] +	Fatty acids	0.988	–	in silico MS/MS match
Fatty acid 7	19.610	318.297	6.026	318.300	C18H39NO3	[M + H] +	Fatty acids	0.965	–	in silico MS/MS match
Fatty acid 9	19.281	288.251	5.738	288.253	C16H33NO3	[M + H] +	Fatty acids	0.997	–	in silico MS/MS match
Terpene 1	20.033	471.267	5.913	471.271	C26H40O6	[M + Na] +	Terpenoids	0.665	–	in silico MS/MS match
Terpene 2	19.200	289.141	3.089	289.144	C17H20O4	[M + H] +	Terpenoids	0.977	–	in silico MS/MS match
Terpene 3	20.189	291.156	6.247	291.159	C17H22O4	[M + H] +	Terpenoids	0.983	–	in silico MS/MS match
Terpene 4	16.2667	293.172	5.060	293.174	C17H24O4	[M + H] +	Terpenoids	0.995	–	in silico MS/MS match
Terpene 5	9.9155	305.135	5.101	305.138	C17H20O5	[M + H] +	Terpenoids	0.994	–	in silico MS/MS match
Terpene 6	15.608	351.178	4.897	351.180	C19H26O6	[M + H] +	Terpenoids	0.995	–	in silico MS/MS match
Terpene 7	19.195	371.143	6.027	371.146	C19H24O6	[M + Na] +	Terpenoids	0.996	–	in silico MS/MS match
Terpene 8	15.621	373.157	4.888	373.162	C19H26O6	[M + Na] +	Terpenoids	0.997	–	in silico MS/MS match
Terpene 9	17.805	751.288	5.719	751.294	C38H48O14	[M + Na] +	Terpenoids	0.966	–	in silico MS/MS match
Terpene 10	18.731	235.167	4.880	235.169	C15H22O2	[M + H] +	Terpenoids	0.986	–	in silico MS/MS match
Terpene 11	10.000	345.128	4.199	345.131	C17H22O6	[M + Na] +	Terpenoids	0.991	–	in silico MS/MS match
Unknown 2	0.581	151.074	2.258	151.075	C9H10O2	[M + H] +	Polyketides	0.630	–	in silico MS/MS match
Unknown 3	0.584	191.066	2.258	191.068	C9H12O3	[M + Na] +	Shikimates and Phenylpropanoids	0.454	–	in silico MS/MS match
Unknown 4	29.645	417.231	8.270	417.227	C24H32O6	[M + H] +	Polyketides	0.884	–	in silico MS/MS match
Unknown 8	22.101	345.172	6.597	345.170	C20H24O5	[M + H] +	Polyketides	0.829	–	in silico MS/MS match
Unknown 11	25.124	383.179	7.384	383.183	C21H28O5	[M + Na] +	Terpenoids	0.741	–	in silico MS/MS match
Unknown 16	24.549	397.195	7.178	397.199	C22H30O5	[M + Na] +	Polyketides	0.805	–	in silico MS/MS match
Unknown 17	29.629	439.205	8.271	439.209	C24H32O6	[M + Na] +	Polyketides	0.783	–	in silico MS/MS match
Unknown 19	14.313	507.180	3.559	507.183	C23H32O11	[M + Na] +	Terpenoids	0.742	–	in silico MS/MS match
Unknown 20	23.123	515.258	6.918	515.263	C27H40O8	[M + Na] +	Terpenoids	0.834	–	in silico MS/MS match
Unknown 21	19.405	531.252	5.905	531.257	C28H36N4O5	[M + Na] +	Amino acids and Peptides	0.944	–	in silico MS/MS match
Unknown 24	0.458	206.044	1.096	206.046	C5H13NO4S	[M + Na] +	Fatty acids	0.465	–	in silico MS/MS match

**Fig. 1:**
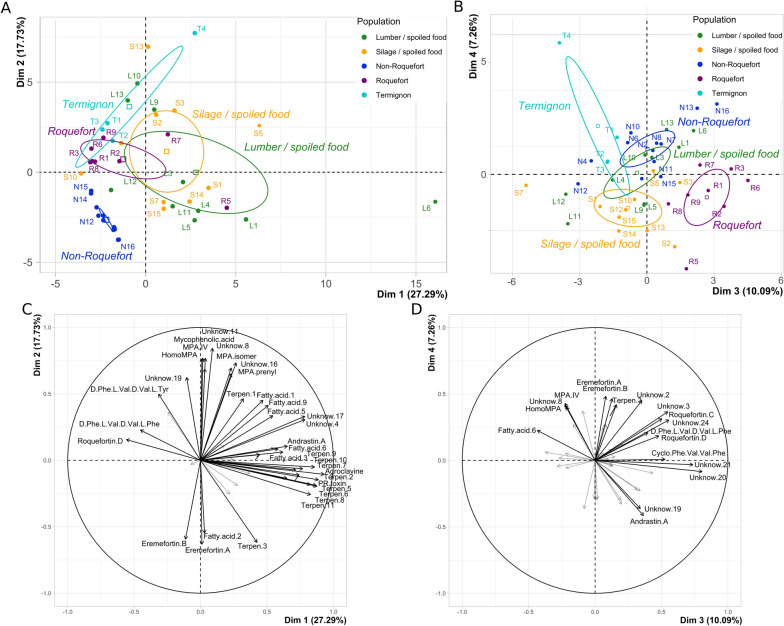
Principal component analysis (PCA) illustrating the metabolite profile differences between *Penicillium roqueforti* populations. **A**. Strains on the first two axes of the PCA. **B**. strains on the third and fourth axes of the PCA. In **A**. and **B**. a confidence ellipse at 95% is drawn for each of the five populations. The percentage of variance explained by the axes are indicated. The same colour code is used as in the other figures: green for the lumber/spoiled food population, orange for the silage/spoiled food population, dark blue for the non-Roquefort cheese population, purple for the Roquefort cheese population and light blue for the Termignon cheese population. The strain IDs are provided in Additional file 1: Table S1. **C**. Association between the two first and **D.** the third and fourth PCA axes and the variables which corresponded to the selected metabolites from metabolite profiling of 44 *P. roqueforti* strains

While some metabolites were produced by all strains in all five *P. roqueforti* populations, others varied across strains, either qualitatively or quantitatively. Principal component analysis (PCA) performed on all 47 metabolites separated the different *P. roqueforti* populations (Fig. 1). The two first dimensions explained 45.94% of the variance, while dimensions 3 and 4 explained 17.5% of the variance. The first dimension separated the cheese populations from the non-cheese populations. Dimension 1 was positively associated with PR toxin, AND A, fatty acids and terpenes, and negatively associated with ROQ C and D, and Phe-Val-Val-Phe. The second PCA dimension mainly separated the non-Roquefort population from the two other cheese populations based on positive associations with ERE A & B, fatty amide 2 and terpene 3 and negative ones with MPA and MPA-associated metabolites (MPA isomer, homo MPA, MPA prenyl and MPA IV), as well as the unknown molecules 4, 8, 11, 16, 17 and 19. The third PCA axis separated the Roquefort populations from all other populations (Fig. 1) and was positively associated with ROQ C, ROQ D and the unknown metabolites 19, 20 and 21. The fourth dimension separated the silage/spoiled food population from the other populations and was positively associated with several fatty acids (*e.g.* 1, 5 and 9).

### Distinct differences in metabolite profiles between cheese and non-cheese populations

**Fig. 2:**
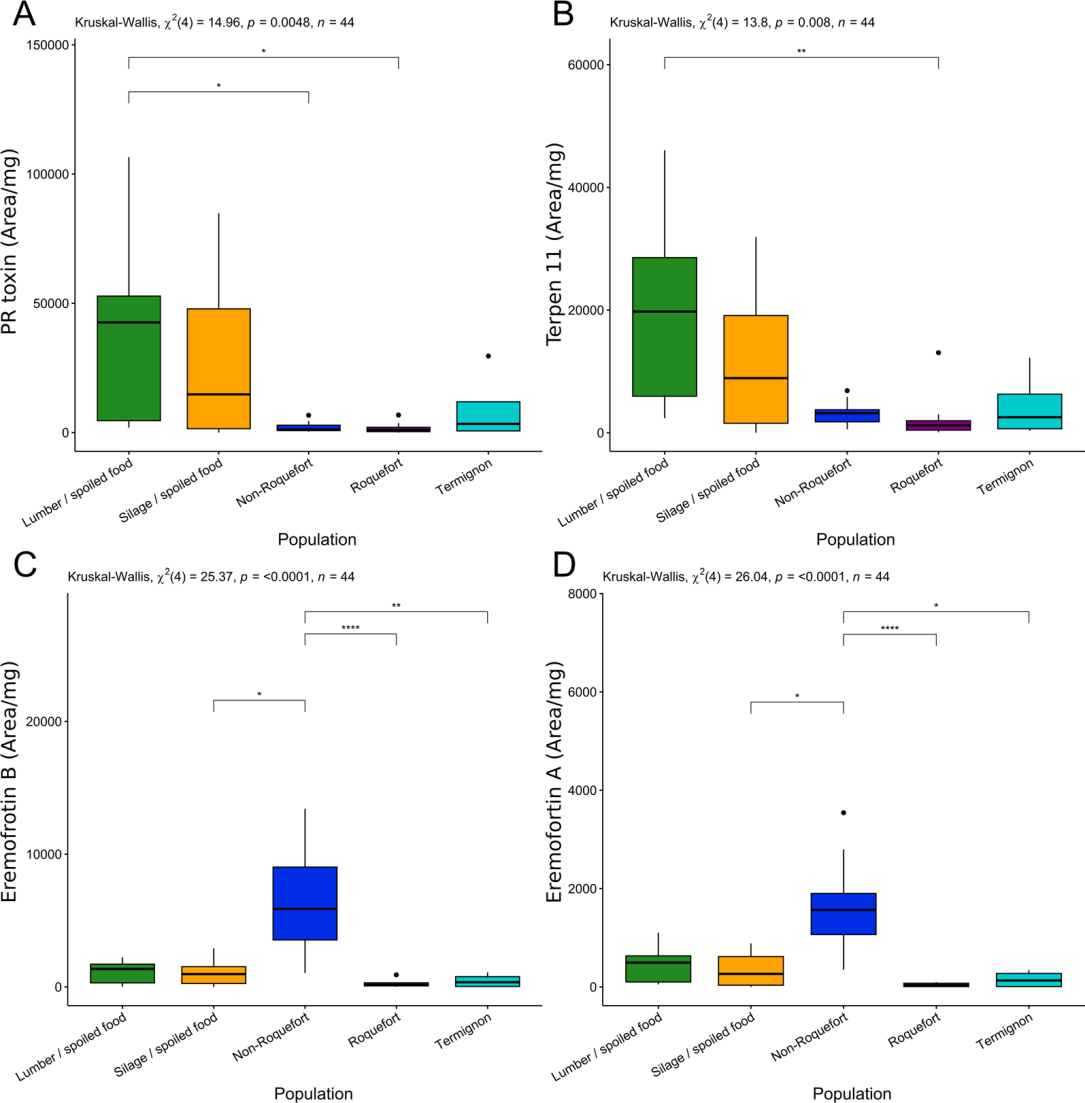
Production level of PR-toxin (**A**), terpene 11 (putative eremofortin C) (**B**), eremofortins A (**C**) and B (**D**) PR toxin intermediates, across the five *Penicillium roqueforti* populations. Production level is expressed as the surface of the peak area of the targeted metabolite per extract mycelium mass. The same colour code is used as in the other figures: green for the lumber/spoiled food population, orange for the silage/spoiled food population, dark blue for the non-Roquefort cheese population, purple for the Roquefort cheese population and light blue for the Termignon cheese population. The results of the statistical test for a population effect is given at the top of each panel. Pairwise significant differences are indicated by asterisks. The boxplots represent the median (center line), the first quartile and third quartile (box bounds), the maximum and minimum excluding outlier points (whiskers), points being the outliers, *i.e.* with values either below the first quartile minus 1.5 fold the interquartile range or above the third quartile plus 1.5 fold the interquartile range

**Fig. 3:**
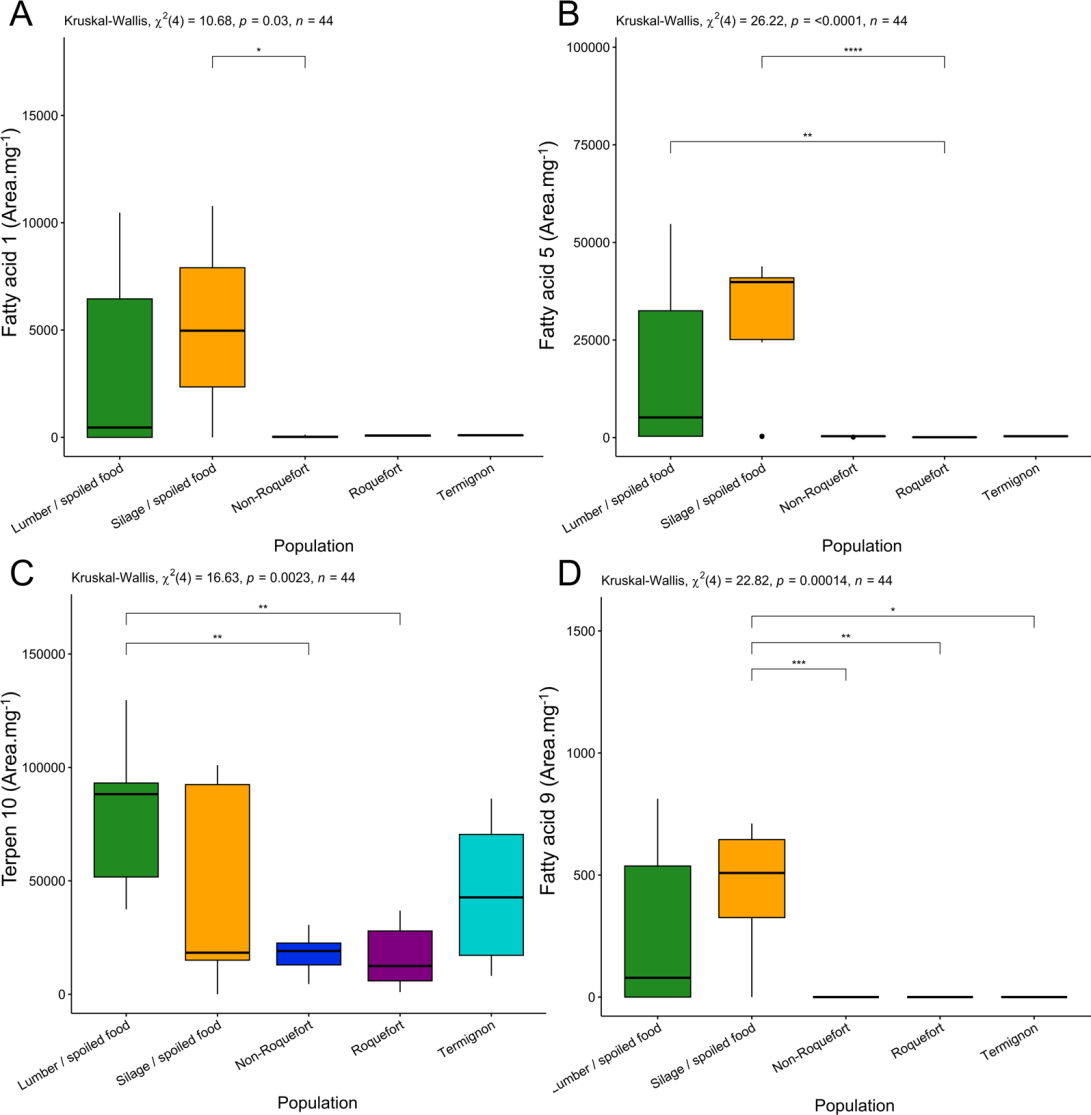
Production levels of the fatty acids 1 (**A**), 5 (**B**), 9 (**D**), and terpene 10 (**C**) across the five *Penicillium roqueforti* populations. Production level is expressed as the surface of the peak area of the metabolite per extract mycelium mass. The different populations were colour-coded as follows: green for the lumber/spoiled food population, orange for the silage/spoiled food population, dark blue for the non-Roquefort cheese population, purple for the Roquefort cheese population and light blue for the Termignon cheese population. The results of the statistical test for a population effect is given at the top of each panel. Pairwise significant differences are indicated by asterisks. The boxplots represent the median (center line), the first quartile and third quartile (box bounds), the maximum and minimum excluding outlier points (whiskers), points being the outliers, *i.e.* with values either below the first quartile minus 1.5 fold the interquartile range or above the third quartile plus 1.5 fold the interquartile range

**Fig. 4:**
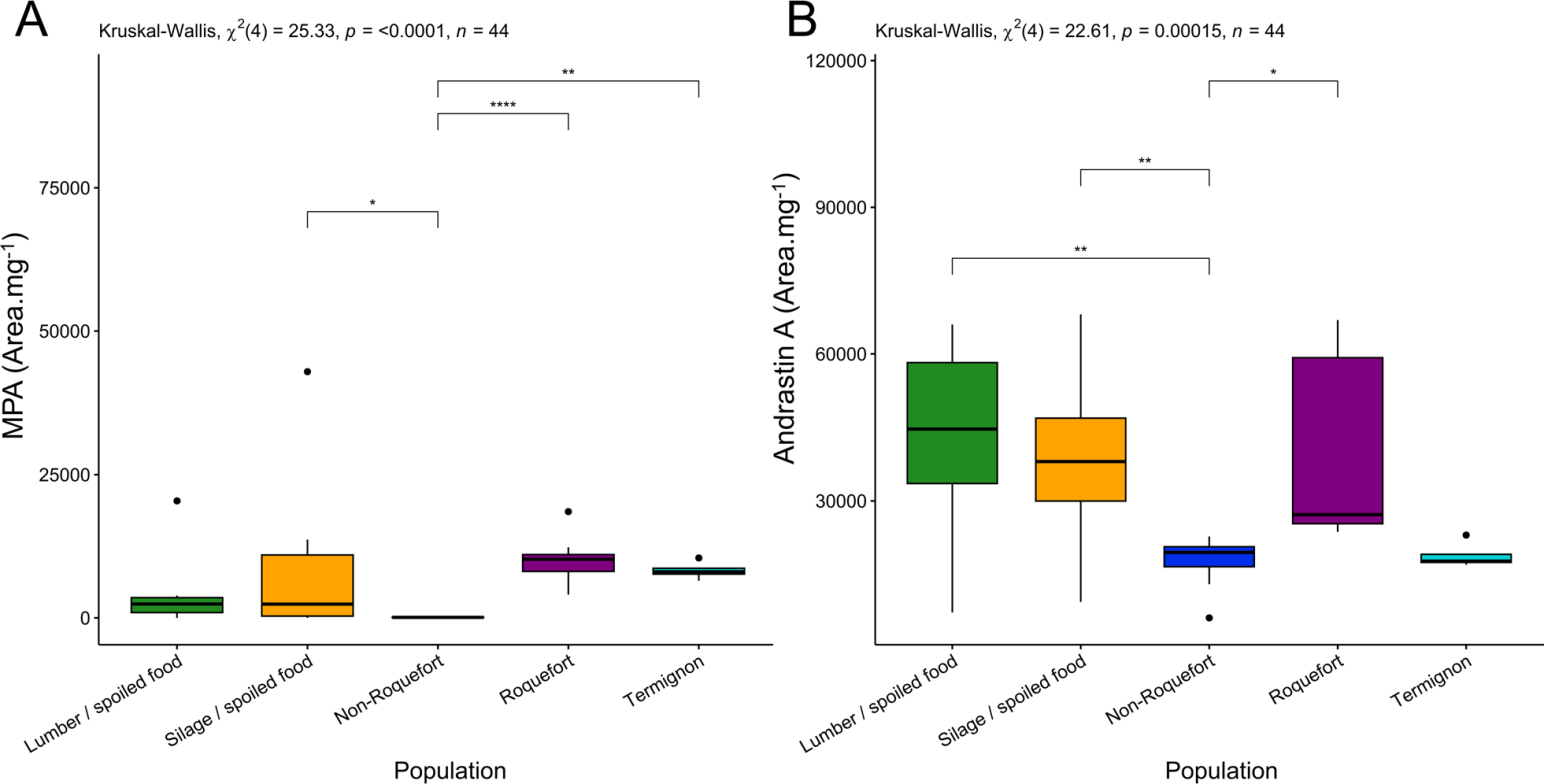
Production level of mycophenolic acid (**A**) and andrastin A (**B**) among the five *Penicillium roqueforti* populations. Production level is expressed as the surface of the peak area of the targeted metabolite per mycelium mass. The different populations were colour-coded as follows: green for the lumber/spoiled food population, orange for the silage/spoiled food population, dark blue for the non-Roquefort cheese population, purple for the Roquefort cheese population and light blue for the Termignon cheese population. The results of the global test for a population effect is given at the top of each panel. Pairwise significant differences are indicated by asterisks. The boxplots represent the median (centre line), the first quartile and third quartile (box bounds), the maximum and minimum excluding outlier points (whiskers), points being the outliers, *i.e.* with values either below the first quartile minus 1.5 fold the interquartile range or above the third quartile plus 1.5 fold the interquartile range

The metabolite profiles were strikingly different between the cheese and non-cheese populations. The most toxic mycotoxin produced by *P. roqueforti,* PR toxin, was produced only by non-cheese strains (except a slight production in the Termignon population) and in highest quantities by the lumber/spoiled food population (Fig. 2A). The non-cheese populations also displayed higher production of the meroterpenoid AND A (Fig. 4B), diverse fatty acids, the isofumigaclavine A intermediate, festuclavine (Additional file 6: Fig S1 B), the unknown compounds 2 and 3 (additional file 7: Fig S2 A & B), the terpenes 5 (Additional file 8: Fig S3 E) and 11 (Fig. 3B), as well as other terpenes. In contrast, cheese populations produced very low or even no detectable amounts of these metabolites (Fig. 2, Additional file 7: Fig S2). In particular, the fatty acids 1, 5 and 9 (Fig. 2A–E) were not detected in cheese strains. The roquefortine C and D alkaloids were in contrast produced in higher quantities in cheese populations than non-cheese populations (Additional file 6: Fig S1 H & I).

### Specific metabolite profiles in each population

The silage/spoiled food population produced the highest levels of two potentially toxic clavines, agroclavine and FUM A (Additional file 6: Fig. S1 A, C), fatty acids 1, 5 and 9 (Fig. 3A). The Roquefort population produced higher levels of ROQ C & D compared to the other cheese populations (Additional file 6: Fig. S1 H & I). The non-Roquefort population produced the lowest levels of the main mycotoxins across all populations; strains from this population nevertheless produced significantly higher quantities of the PR toxin intermediates, ERE A & B, than the other populations (Fig. 2B, C), suggesting that the PR toxin pathway was partially functional. Only low levels of each targeted metabolite of the PR toxin pathway could be detected in the Roquefort population, a single strain producing quantifiable amounts of a single eremofortin (ERE B for LCP02939; Additional file 3: Table S3). This suggests that the PR toxin production pathway may be non-functional or down-regulated.

We detected very low quantities of mycophenolic acid across all populations compared to other quantified metabolites, maximal concentrations only reaching 118 ng.g^-1^.mg^-1^. Moreover, 43% of tested strains did not produce any detectable amount of mycophenolic acid, in particular all non-Roquefort strains (Fig. 4A). In contrast, all Roquefort and Termignon strains produced detectable MPA levels (Fig. 4A). Only Termignon strains produced significant levels of MPA-related metabolites, including identified compounds (MPA isomer, homo MPA, MPA prenyl and MPA IV; Additional file 6: Fig. S1 D, E, F and G) as well as unknown compounds (unknown 4 and 8; Additional file 7: Fig S2 C & D). The non-Roquefort and Termignon populations produced low levels of AND A, while the Roquefort population produced higher amounts, although in variable quantities (Fig. 4B).

Each cheese population also exhibited metabolite specificities in terms of fatty acids and terpenes. The non-Roquefort population produced high levels of fatty acid 2 and terpene 3, and also the highest amounts of the unknown metabolite 24 which harbours a sulfur (additional file 7: Fig S4D, S3C and S2K, respectively); sulfur-containing specialised metabolites are often associated with bioactive properties. The Roquefort population produced high levels of unknown metabolites 19, 20 and 21 (Additional file 7: Fig S2H, I and J), while the Termignon strains produced at higher levels the unknown metabolites 4, 8, 11 and 16 (Additional file 7: Fig S2 C, D, H, I and J).

### Genetic specificities explain MPA and PR-toxin production differences

**Fig. 5:**
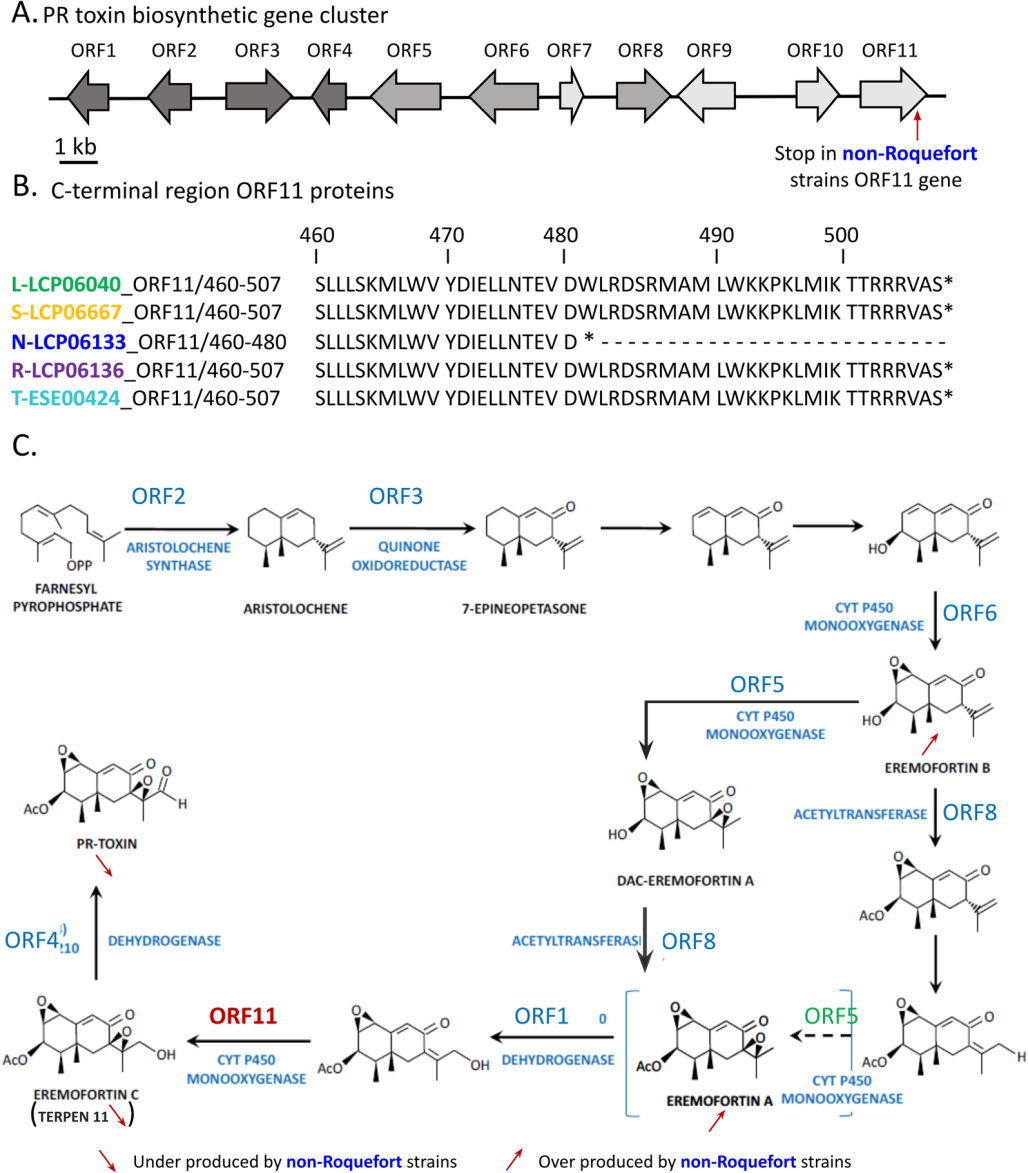
PR toxin biosynthetic cluster and pathway in *Penicillium roqueforti*. (**A**) PR toxin biosynthetic gene cluster in *Penicillium roqueforti* as described in Hidalgo et al (2017). Genes silenced in Hidalgo et al. (2014) are in dark grey, genes silenced in Hidalgo et al (2017) and untargeted genes are in light grey. (**B**) C-terminal region of ORF11 protein in strains representing their population, “lumber/ spoiled food” in green, “silage/spoiled food” in orange, “non-Roquefort” in blue, “Roquefort” in purple, and “Termignon” in light blue. (**C**) Proposed position for the intervention of ORF11 (red) protein in the PR-toxin production pathway. Pathway figure adapted from Chàvez et al. (2023). Position of ORF 5 is not the one proposed in Hidalgo et al (2017) but coherent with an under production of eremofortin A and PR toxin, and an overproduction of eremofortin B observed when the gene is silenced

The genomes of the 44 studied strains were analysed to compare their biosynthetic gene clusters in order to understand the observed differences in terms of PR toxin and MPA production. For the MPA biosynthetic gene cluster, genomic comparisons showed that all strains from the non-Roquefort population, producing no MPA, exhibited a 174 bp deletion in the *mpaC* gene. The deletion is situated in the lipase/esterase domain of the MpaC enzyme, which is the key polyketide synthase enzyme of this cluster (Gillot et al. 2017a). Indeed, this nonreducing polyketide synthase catalyses the synthesis of the first reaction intermediate, 5-methylorsellinic acid (5-MOA) from acetyl-CoA, 3 malonyl-CoA and S-adenosylmethionine (Regueira et al. 2011). This deletion in the 3’ region of the gene introduces a frameshift in the translated protein, leading to a truncated protein (2477 aa) compared to the normal-sized protein (2491 aa) of the MpaC sequence found in the other populations (Fig. 5A). In addition to the non-Roquefort strains, seven other strains did not produce quantifiable levels of MPA (three out of ten strains from the lumber/spoiled food population and four out of ten strains from the silage/spoiled food populations). However, no *mpaC* gene deletion or other modifications affecting the gene cluster were detected in their genomes.

The genomic comparison of the PR toxin biosynthetic gene clusters revealed that all studied non-Roquefort strains presented a G-to-A substitution in position 1440 of ORF 11, which codes for a cytochrome P450 monooxygenase (Fig. 5B). This substitution introduces a premature stop codon in the place of a tryptophan codon, resulting in a truncated version of the enzyme, with 27 aa missing in the 3’ region, leading to a protein of 480 aa instead of 507 aa. The truncation only present in non-Roquefort strains, together with their lack of PR toxin production and the accumulation of the ERE A & B intermediates, suggests that the truncated enzyme is not fully functional. In the Roquefort population, low PR toxin was produced and ERE A & B did not accumulate, but the PR toxin biosynthetic gene cluster displayed no differences with the functional gene clusters in the other *P. roqueforti* populations (Fig. 5B). The lack of PR toxin production and of its intermediates may thus be due to *trans*-down-regulation.

A premature stop codon was also observed in the *ifgB* gene of 10 strains from silage, lumber/spoiled food and Termignon population, resulting in a truncated protein of 338 instead of 340 amino acids. However, no lower production of FUM A was observed for the concerned strains compared to others. A frameshift was observed in the *ifgI* gene of the LCP06040 strain from lumber/spoiled food population, resulting in a truncated protein of 304 instead of 384 amino acids. Interestingly, LCP06040 was the only *P. roqueforti* strain that did not produce FUM A. Concerning the roquefortine C biosynthesis gene cluster, the 5’ region of the *rds* gene had a deletion leading to the absence of a start codon in UBOCC-A-118017 and UBOCC-A-118018 from the silage population, as well as a frameshift due to a deletion in the strain LCP02939 from the Roquefort population. These three strains, LCP02939, UBOCC-A-118017 and UBOCC-A-118018, were the only strains which did not produce any roquefortine C. However, none of the mutations leading to a truncated gene cluster for roquefortine C or FUM A were linked to a given population. For AND A, while sequence variations were observed in some strains, these variations did not introduce a truncated gene and were shared by several populations.

## Discussion

In this study, we compared metabolite production patterns between strains belonging to the five known *P. roqueforti* populations. We found that the domesticated Roquefort and non-Roquefort cheese populations produced fewer metabolites and were less mycotoxinogenic than their non-cheese counterparts. These findings provide a more thorough understanding on the population divergence and domestication history of *P. roqueforti.* The lower toxin production levels in domesticated populations may be due to selection for healthier cheeses, or to relaxed selection (i.e. the reduction in the strength of purifying selection due to a function being less used), if toxins are not useful any more in the cheese environment compared to wild or other anthropized environments (Lu et al. 2006; Rokas 2009).

To understand adaptation and specialisation in contrasting ecological niches, comparing phenotypes of strains associated with different environments is essential to identify adaptive traits. In fungi, the phenotype comparison approaches include estimating the growth cardinal values (*e.g.* temperature, pH or *a*_*w*_), ability to use different substrates and resistance to toxic compounds as recently done for example in *P. roqueforti* (Dumas et al. 2020; Crequer et al. 2023) and in the rice blast fungus *Pyricularia oryzae* (Thierry et al. 2022). Comparisons of metabolite production profiles, especially patterns of specialised metabolites, are of major interest in this context as metabolites can be involved in a wide range of biotic interactions and abiotic responses, which can significantly impact fitness and confer competitive advantages.

In this study, we analysed 44 strains from the five known *P. roqueforti* populations (Dumas et al. 2020; Crequer et al. 2023), using a metabolomics approach to compare their metabolite profiles after growth on YES medium and genomic comparison of their metabolite biosynthetic gene clusters. An ANOVA analysis of the average dry biomass for each strain, grouped by population, showed no significant difference between populations at day 10 (Table S5). This indicated that there was no notable difference in growth between populations. Beyond the seven targeted secondary metabolites, which included the main known *P. roqueforti* mycotoxins, 40 other fungal metabolites were identified (Table 1). The metabolite production profiles were different between the five *P. roqueforti* populations*,* and in particular between the cheese and non-cheese populations, which we could explain for some mycotoxins by deletions in genes involved in their biosynthesis in the non-Roquefort population.

A major finding relates to the PR toxin, the most toxic known *P. roqueforti* metabolite, with significant differences of production levels between non-cheese and cheese populations. The non-cheese populations produced, on average, higher concentrations of PR toxin, especially the Lumber/spoiled food population, while the non-Roquefort and Roquefort domesticated populations did not produce any quantifiable quantities. The Termignon population did produce some PR toxin, but at a lower level than the non-cheese populations. Such an intermediate profile of the Termignon population, between the cheese and non-cheese populations, has previously been reported for various growth parameters and carbon source usage (Crequer et al. 2023). The intermediate metabolite production levels are consistent with the hypothesis that the Termignon population represents descendants of an ancestral domesticated population, displaying traits resulting from domestication before the strong selection imposed in recent years by process industrialisation, thus corresponding to a protracted domestication process (i.e. a slow process occurring across hundreds or thousands of years), as reported in several crops (Allaby et al. 2008; Fuller et al. 2012). The recent study on *P. roqueforti* isolates from non-inoculated Turkish cheeses (Kirtil et al. 2024) could further provide information on this species domestication process as some of these characterized strains were closely related to the Termignon population. Regarding the two populations used for cheese inoculation, non-Roquefort and Roquefort, our results are of particular interest for food safety and human health, as these populations did not produce PR toxin which is the most toxic *P. roqueforti* mycotoxin (Pedrosa & Griessler 2010; Hymery et al. 2017).

On the other hand, *P. roqueforti* is one of the most common post-harvest fungal contaminants in silages (Gallo et al. 2015). Its ability to colonise this substrate, and produce there PR toxin, has been associated with cattle intoxication, with symptoms such as loss of appetite, cessation of rumen activity, gastroenteritis, haemorrhage and even death (Veselý et al. 1981; Nielsen et al. 2006). It was considered so far that the *P. roqueforti* strains used as ripening cheese cultures had the intrinsic ability to produce PR toxin (Dubey et al. 2018) and that the absence of this mycotoxin in cheeses was due to its instability and presumed degradation into various less toxic molecules, i.e. PR imine and PR amide and PR acid (Chang et al. 1993, 1996). While the latter hypothesis may still be valid, our results provide a new and robust explanation, as none of the studied non-Roquefort and Roquefort strains, used for blue cheese production, produced quantifiable levels of PR toxin, even in YES medium known to be favourable for secondary metabolite production. Cheeses made with potential PR-toxin producer strains, such as the Termignon population, may nevertheless contain little of this toxin in cheeses as it is unstable in this matrix. Indeed, PR toxin was shown to be degraded in PR amine in cheese (Siemens & Zawistowski 1993). The instability of PR toxin in cheese may imply that the loss of its production ability in cheese strains may be more due to relaxed selection than to selection against its production in cheeses.

Diversity in metabolite synthesis, both qualitatively and quantitatively, may arise from divergent selection across ecological niches. Contrasting metabolite profiles across differentiated fungal populations or lineages of a given species have been reported in other fungi. For example, among the four lineages identified in *Fusarium graminearum* isolated from maize (Lee et al. 2012), most isolates from lineages 2 and 6 produced the trichothecene group B mycotoxin nivalenol (NIV), while all isolates from lineages 3 and 7 produced deoxynivalenol (DON), another major trichothecene B *Fusarium* mycotoxin, that is a virulence factor in wheat and toxic for human and animal health. In *Fusarium asiaticum* isolated from Chinese rice and wheat, producer strains of 3-acetyldeoxynivalenol (an acetylated form of DON) were ubiquitous in wheat while NIV-producers were more prevalent in rice, the trichothecene chemotypes also varying across regions (Yang et al. 2018). In *Aspergillus flavus,* the production of aflatoxin B1 (AFB1), a potent cancerogenic mycotoxin regulated in the food chain, was significantly higher for soil isolates than for corn kernel ones (Sweany et al. 2011).

To further understand the differences in metabolite production between *P. roqueforti* populations, we focused on determining the genetic basis for two main differences between cheese and non-cheese populations, i.e. the production of PR toxin and MPA. The inability to produce PR toxin by non-Roquefort strains could be attributed to a substitution in ORF 11 of the corresponding biosynthetic gene cluster resulting in a premature stop codon. This finding, combined with the accumulation of both ERE A & B, suggests that ORF 11 likely intervenes in the formation of eremofortin C (ERE C), the final precursor for PR toxin, instead of ORF 5 as previously described (Hidalgo et al. 2017). Our results also pinpointed an unknown metabolite that may correspond to ERE C (terpene 11); this metabolite was not found in the non-Roquefort population extracts either, which reinforces the hypothesis that ORF 11 intervenes in its formation. However, as no commercial ERE C standard was available, we could not fully confirm the identity of terpene 11 nor test the possibility that these isolates can produce PR toxin from ERE C.

The Roquefort population produced no detectable amount of PR toxin, ERE A, ERE B or putative ERE C (*i.e.* terpene 11), and we were unable to determine the genetic basis of this lack of production based on biosynthetic gene cluster comparisons. It seems most likely that the expression of the entire gene cluster might be affected by a regulatory element in *cis* or *trans*. In the DS17690 *P. chrysogenum* strain, downregulation of the PR toxin biosynthetic cluster was due to mutations in the *laeA* and *velA* regulatory genes (Martín 2017). Here, we did not identify any mutations in either of these two genes in the Roquefort strains (data not shown); therefore, the inability to produce PR toxin may be due to identified global regulators (*e.g. pga1, sfk1, pcz1*) involved in the modulation of metabolite production in *P. roqueforti* (Chávez et al. 2023) or to other, unidentified regulators.

None of the non-Roquefort strains produced mycophenolic acid and we could attribute this inability to a deletion in the *mpaC* gene within the corresponding biosynthetic gene cluster. This deletion had been previously reported in *P. roqueforti* strains (Gillot et al. 2017a), and found associated with the presence of the horizontally transferred *CheesyTer* and *Wallaby* regions (Gillot et al. 2017b), that were later found mostly present in the non-Roquefort population (Dumas et al. 2020). A recent study using samples from Turkish moldy blue cheese (Kirtil et al. 2024) also indicated that this deletion appears to be present only in the non-Roquefort population. It also confirmed the presence of the *CheesyTer* and *Wallaby* regions in the studied strains as also shown in the Termignon population (Crequer et al. 2023). The Termignon strains were the strains presenting the highest production of MPA and MPA-related derivatives, which is of interest for large-scale production of this important pharmaceutical immunosuppressive with antifungal, antibacterial, antiviral, anti-psoriasis and antitumor and anti-graft reject activities (Ammar et al. 2023).

In *P. roqueforti,* other specialised metabolites were produced by all populations, but with still marked differences in production levels for several compounds. For the ROQ C & D alkaloids, the highest producers were found in the cheese populations, especially the Roquefort population. The fact that ROQ C & D production was maintained in domesticated populations raises questions about their ecological role in cheese, given that roquefortines have various bioactive properties. It may also be that there was no selection during domestication against the production of this specialised metabolite with low cytotoxic effects (Fontaine et al. 2016).

We also identified differences between *P. roqueforti* populations for the production of clavines, *e.g.* FUM A, festuclavine (a FUM A intermediate) and agroclavine. The production of festuclavine and agroclavine by *P. roqueforti* had previously been reported but not compared between populations (Ohmomo et al. 1975; Nielsen et al. 2006). We found the smallest quantities of clavine in the Termignon and non-Roquefort cheese populations. Similar results were also observed for andrastin A, a potential natural anti-cancer compound, thus raising the question of the ecological role of this molecule, especially in cheese populations.

Numerous other untargeted molecules were observed and corresponded to terpenoids, fatty acids (including fatty acid amides) or unidentified molecules. These molecules might correspond to metabolites recently described in *P. roqueforti,* such as annullatins (Xiang et al. 2022), eremophilane and guaiane sesquiterpenes (Mo et al. 2023) or sesterterpenoids (Wang et al. 2018, 2020, 2021), their role and biological activity being still unknown. Further efforts are required to refine their identification and understand their function. Several molecules were specific to some populations, *e.g.* unknown compounds 2 and 3, and terpene 11 specific to the lumber population and fatty acid 5 specific to the silage/spoiled food population, terpene 3 specific to the non-Roquefort population, unknown compounds 20 and 21 specific to Roquefort population, and unknown compounds 8 and 11 specific to the Termignon population; they could thus be of clear interest as potential population biomarkers and for being involved in niche specialisation.

The non-Roquefort population, which displays the strongest domestication syndrome and the most severe genetic bottleneck (Dumas et al. 2020; Crequer et al. 2023), also exhibited the most distinctive metabolite profile. The non-Roquefort strains produced the lowest amounts of metabolites for both identified compounds (including mycotoxins) or unidentified compounds. Such toxin loss represents a convergence in domesticated fungal populations, and may result, as previously mentioned, from a neutral degeneration of unused traits or a selection against toxin production by humans. Fungal metabolites are known to be used for microbial “chemical warfare”, *e.g.* for fungal invasion in plants and/or microbial competition, so they might not be required to the same extent in a rich medium with readily available nutrients and with an inoculation advantage for matrix colonisation. We can hypothesize that this is the result of either the degeneration of the toxinogenic trait in domesticated populations due to a relaxation of the purifying selection (i.e. if the production of these metabolites is not beneficial in cheese) or the selection which likely occurred through practical observations in cheese production. Strains producing lower levels of mycotoxins may have been favoured as they might have contributed to better cheese quality, either in terms of organoleptic traits, shelf-life or or less deleterious physiological effects. Other unused traits have been reported to degenerate in domesticated fungi by relaxed selection, such as the ability of carbohydrate use and of sexual reproduction (Ropars et al. 2016; Ropars and Giraud 2022). Genomic studies have reported that *P. roqueforti* harbors a non-functional gene cluster for the production of the toxic mycotoxin patulin, notably due to the absence or truncation of several genes, including *patE* and *patF* (Nielsen et al. 2017, Yin et al. 2021, Garello et al. 2024). These genes are essential for patulin biosynthesis in *P. expansum* (Li et al. 2019). However, patulin production has been reported in *P. roqueforti* strains isolated from Turkish cheeses (Erdogan et al. 2003, Cakmakci et al. 2015), suggesting that further comparative studies on patulin production and its biosynthesis gene cluster could be of interest. In the present study, genomic comparisons allowed identifying the mutations likely causing loss of production of the PR toxin and MPA in the non-Roquefort population.

The reduced mycotoxinogenesis can indeed be the result of domestication events, as humans have often selected fungal strains unable to produce harmful toxins for use in food. The best known example is *Aspergillus oryzae,* a domesticated species used to ferment Asian food products derived from its mycotoxin-producing wild relative, *Aspergillus flavus* (Barbesgaard et al. 1992). In *A. oryzae*, several mutations were reported in the aflatoxin biosynthetic gene cluster, in particular an approximately 40 kb deletion in the genomic region between the *norB* and *norA* genes (Chang et al. 2005), mutations in the *aflR* promoter, a nearly 250 bp deletion in the *aflT* coding region, a frameshift mutation in the *norA* coding region and multiple nonsynonymous mutations in the *verA* coding region (Tominaga et al. 2006). Down-regulation of another mycotoxin, cyclopiazonic acid (CPA), also occurs in *A. oryzae* (Gibbons et al. 2012). Another example is the domesticated fungus *Aspergillus sojae,* a species considered to be derived from *Aspergillus parasiticus* (Chang and Hua 2023). In *A. sojae,* the inability to produce aflatoxin is the result of a termination point mutation in the *aflR* regulatory gene as well as a premature stop codon in the *pksA* gene leading to a truncated version of the polyketide synthase enzyme (Chang et al. 2007). Another example, among *Penicillium* species, is *P. camemberti*, also domesticated for cheese making*.* In this species, two different lineages display very contrasted mycotoxin production profiles: *P. camemberti* var. *camemberti* produces high levels of cyclopiazonic acid (CPA) on YES medium while *P. camemberti* var. *caseifulvum* does not. This was shown to be due to a 2-bp deletion in the *cpaA* gene, inducing a frameshift, thus modifying the polyketide synthase/non-ribosomal peptide synthase enzyme responsible for the first step of the CPA biosynthetic pathway (Ropars et al. 2020).

## Conclusion

To conclude, a dual targeted and untargeted metabolomics approach was used to compare *P. roqueforti* metabolite profiles. Distinct profiles were identified across the five *P. roqueforti* populations, which is likely due to ecological specialisation and human selection. Indeed, the two domesticated populations used to inoculate blue cheeses no longer produce PR toxin, the most toxic *P. roqueforti* mycotoxin, while the Termignon strains produce low levels*.* In contrast, the non-cheese populations (Lumber/spoiled food and Silage) maintained their PR toxin production which indicates that this mycotoxin likely plays an important ecological role in these more complex and harsh environments where microbial competition and natural colonisation occurs, although its precise role remains unknown. The metabolite diversity and quantity profile is unique to each *P. roqueforti* population and likely provides specific advantages to thrive in their respective complex domesticated or wild environments. To further explore such an adaptive role, it would be of interest to determine the production of these metabolites for each population in media more closely resembling the composition of their respective ecological niches, such as cheese, silage and wood. Overall, this study provides new findings supporting that fungal metabolite profiles are a result of adaptation to contrasting environmental conditions (*i.e.* niche specialisation) and that domestication leads to hypotoxigenic populations.

## Supplementary Information

**Additional file 1: Table S1**: *Penicillium roqueforti* strains used for metabolite profiling: with their IDs, assigned genetic population, sampling origin and date, genome accession number when available and associated reference.

**Additional file 2: Table S2**: Method performance characteristics for metabolite quantification in YES medium. RT: Retention time; R2: determination coefficient; DL: Detection limit; QL: Quantification limit; ESI: Electrospray ionization; NA: not applicable.

**Additional file 3: Table S3**: Targeted metabolite concentrations (ng of metabolite /g of extracted medium / mg of mycelium) and measured mycelium mass in YES medium after 10 days’ culture at 25°C for each screened isolate. For PR toxin concentration is expressed in area /g of extracted medium /g of mycelium as no quantifiable standard was available.

**Additional file 4: Table S4**: Metabolite area.mg-1 of mycelium in YES medium after 10 days day culture at 25°C for each screened isolate.

**Additional file 5: Table S5**: Statistical result of comparison of *Penicillium roqueforti* populations production in metabolites. A. Kruskall-Wallis test results for data with non normal distribution C. Dunn tests results B. ANOVA test results and D. Tukey tests for data with normal distribution. A squared 2 transformation was made on data for eremofortins A & B.

**Additional file 6: Figure S1**: Production level of agroclavine (A), festuclavine A (B), Isofumigaclavine A (C), mycophenolic acid (MPA) isomer (D), homo-MPA (E), MPA-IV (F), -MPA-prenyl (G), roquefortine C and (H) roquefortine D (I) among the five *Penicillium roqueforti* populations. Production level is expressed as the surface of the peak area of the targeted metabolite per extract matrix mass and mycelium mass. The different populations were colour-coded as follows: green for the lumber/spoiled food population, orange for the silage/spoiled food population, dark blue for the non-Roquefort cheese population, purple for the Roquefort cheese population and light blue for the Termignon cheese population. The results of the global test for a population effect is given at the top of each panel. Pairwise significant differences are indicated by asterisks. The boxplots represent the median (centre line), the first quartile and third quartile (box bounds), the maximum and minimum excluding outlier points (whiskers), points being the outliers, i.e. with values either below the first quartile minus 1.5 fold the interquartile range or above the third quartile plus 1.5 fold the interquartile range.

**Additional file 7: Figure S2**: Production level of unknown 2 (A), unknown 3 (B), unknown 4 (C), unknown 8 (D), unknown 11 (E), unknown 16 (F) unknown 17 (G), unknown 19 (H), unknown 20 (I), unknown 21 (J), unknown 24 (K) with formulas in Table 1, among the five *Penicillium roqueforti* populations. Production level is expressed as the surface of the peak area of the targeted metabolite per extract matrix mass and mycelium mass. The different populations were colour-coded as follows: green for the lumber/spoiled food population, orange for the silage/spoiled food population, dark blue for the non-Roquefort cheese population, purple for the Roquefort cheese population and light blue for the Termignon cheese population. The results of the global test for a population effect is given at the top of each panel. Pairwise significant differences are indicated by asterisks. The boxplots represent the median (centre line), the first quartile and third quartile (box bounds), the maximum and minimum excluding outlier points (whiskers), points being the outliers, i.e. with values either below the first quartile minus 1.5 fold the interquartile range or above the third quartile plus 1.5 fold the interquartile range.

**Additional file 8: Figure S3**: Production level of terpene 1 (A), terpene 2 (B), terpene 3 (C), terpene 4 (D), terpene 5 (E), terpene 6 (F), terpene 7 (G), terpene 8 (H), terpene 9 (I) with formulas in Table 1, among the five *Penicillium roqueforti* populations . Production level is expressed as the surface of the peak area of the targeted metabolite per extract matrix mass and mycelium mass. The different populations were colour-coded as follows: green for the lumber/spoiled food population, orange for the silage/spoiled food population, dark blue for the non-Roquefort cheese population, purple for the Roquefort cheese population and light blue for the Termignon cheese population. The results of the global test for a population effect is given at the top of each panel. Pairwise significant differences are indicated by asterisks. The boxplots represent the median (center line), the first quartile and third quartile (box bounds), the maximum and minimum excluding outlier points (whiskers), points being the outliers, i.e. with values either below the first quartile minus 1.5 fold the interquartile range or above the third quartile plus 1.5 fold the interquartile range.

**Additional file 9: Figure S4**: Production level of cyclo-(Phe-Val-Val-Phe) (A), Phe-Val-Val-Phe (B), Phe-Val-Val-Tyr (C), fatty acid 2 (D), fatty acid 3 (E), fatty acid 6 (F), among the five *Penicillium roqueforti* populations. Production level is expressed as the surface of the peak area of the targeted metabolite per extract matrix mass and mycelium mass. The different populations were colour-coded as follows: green for the lumber/spoiled food population, orange for the silage/spoiled food population, dark blue for the non-Roquefort cheese population, purple for the Roquefort cheese population and light blue for the Termignon cheese population. The results of the global test for a population effect is given at the top of each panel. Pairwise significant differences are indicated by asterisks. The boxplots represent the median (center line), the first quartile and third quartile (box bounds), the maximum and minimum excluding outlier points (whiskers), points being the outliers, i.e. with values either below the first quartile minus 1.5 fold the interquartile range or above the third quartile plus 1.5 fold the interquartile range.

## Data Availability

The genome datasets generated during and/or analysed during the current study are available in GenBank via the accession numbers provided. All other datasets generated during and/or analysed during the current study are available from the corresponding author on reasonable request.

## Abbreviations

ACN: Acetonitrile
AFB1: Aflatoxin B1
AND A: Andrastin A
CDS: Coding DNA Sequences
CPA: Cyclopiazonic acid
DON: Deoxynivalenol
DMSO: Dimethyl sulfoxide
ESI: Electrospray ionization
ERE A: Eremofortin A
ERE B: Eremofortin B
ERE C: Eremofortin C
ESE: Ecology Systematics and Evolution
EU: European Union
FUM A: (Iso)-fumigaclavine A
GNPS: Global natural product social molecular networking
HR: High resolution
LC-Q-TOF: High Performance Liquid Chromatography Quadrupole Time-of-Flight Mass Spectrometry
MGF: Mascot generic format
MPA: Mycophenolic acid
MS: Mass spectrometry
NIV: Nivalenol
ORF: Open reading frame
PDA: Potato dextrose agar
PDO: Protected designation of origin
ROQ C: Roquefortine C
ROQ D: Roquefortine D
UBOCC: Université de Bretagne Occidentale Culture Collection
UHPLC-DAD-Q-TOF–MS/MS: Ultra-high-performance liquid chromatography Diode array detection—quadrupole time of flight mass spectrometry
YES: Yeast extract sucrose medium
