# Supplementary figures and images for: Different metabolite profiles across Penicillium roqueforti populations associated with ecological niche specialisation and domestication
Source: IMA Fungus. 2024 Nov 28;15:38. doi: 10.1186/s43008-024-00167-4 (PMC11605963; doi:10.1186/s43008-024-00167-4)

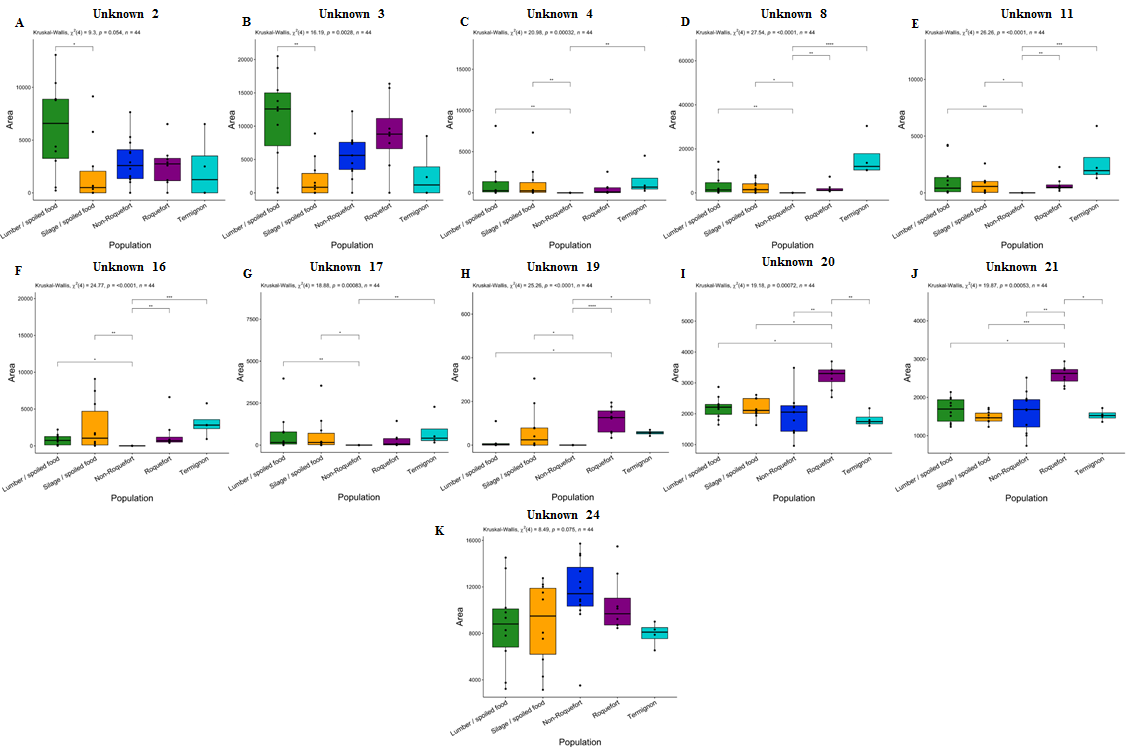

Supplement: Supplementary file 7 — Additional file 7: Figure S2: Production level of unknown 2 (A), unknown 3 (B), unknown 4 (C), unknown 8 (D), unknown 11 (E), unknown 16 (F) unknown 17 (G), unknown 19 (H), unknown 20 (I), unknown 21 (J), unknown 24 (K) with formulas in Table 1, among the five Penicillium roqueforti populations. Production level is expressed as the surface of the peak area of the targeted metabolite per extract matrix mass and mycelium mass. The different populations were colour-coded as follows: green for the lumber/spoiled food population, orange for the silage/spoiled food population, dark blue for the non-Roquefort cheese population, purple for the Roquefort cheese population and light blue for the Termignon cheese population. The results of the global test for a population effect is given at the top of each panel. Pairwise significant differences are indicated by asterisks. The boxplots represent the median (centre line), the first quartile and third quartile (box bounds), the maximum and minimum excluding outlier points (whiskers), points being the outliers, i.e. with values either below the first quartile minus 1.5 fold the interquartile range or above the third quartile plus 1.5 fold the interquartile range. [file 43008_2024_167_MOESM7_ESM.png]

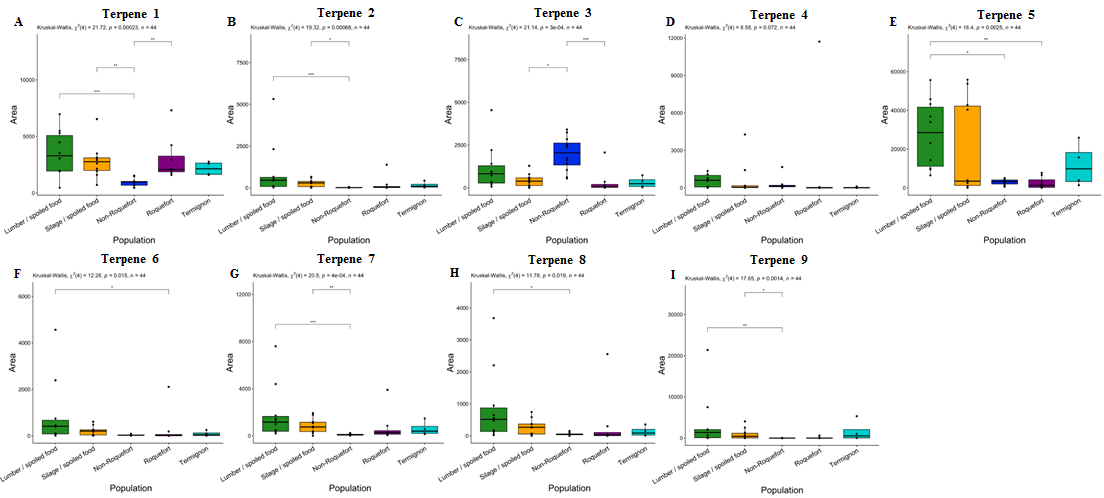

Supplement: Supplementary file 8 — Additional file 8: Figure S3: Production level of terpene 1 (A), terpene 2 (B), terpene 3 (C), terpene 4 (D), terpene 5 (E), terpene 6 (F), terpene 7 (G), terpene 8 (H), terpene 9 (I) with formulas in Table 1, among the five Penicillium roqueforti populations . Production level is expressed as the surface of the peak area of the targeted metabolite per extract matrix mass and mycelium mass. The different populations were colour-coded as follows: green for the lumber/spoiled food population, orange for the silage/spoiled food population, dark blue for the non-Roquefort cheese population, purple for the Roquefort cheese population and light blue for the Termignon cheese population. The results of the global test for a population effect is given at the top of each panel. Pairwise significant differences are indicated by asterisks. The boxplots represent the median (center line), the first quartile and third quartile (box bounds), the maximum and minimum excluding outlier points (whiskers), points being the outliers, i.e. with values either below the first quartile minus 1.5 fold the interquartile range or above the third quartile plus 1.5 fold the interquartile range. [file 43008_2024_167_MOESM8_ESM.png]

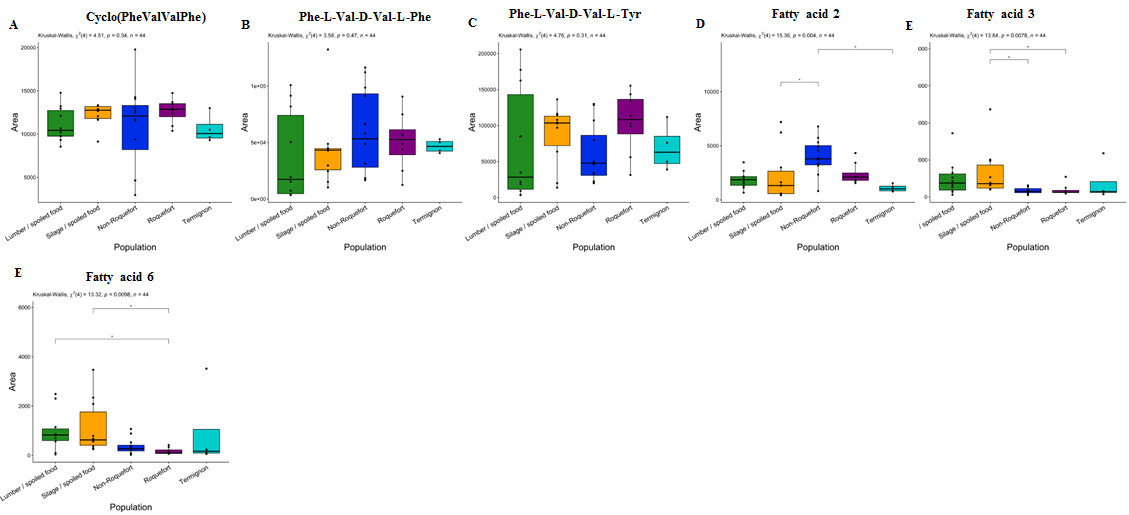

Supplement: Supplementary file 9 — Additional file 9: Figure S4: Production level of cyclo-(Phe-Val-Val-Phe) (A), Phe-Val-Val-Phe (B), Phe-Val-Val-Tyr (C), fatty acid 2 (D), fatty acid 3 (E), fatty acid 6 (F), among the five Penicillium roqueforti populations. Production level is expressed as the surface of the peak area of the targeted metabolite per extract matrix mass and mycelium mass. The different populations were colour-coded as follows: green for the lumber/spoiled food population, orange for the silage/spoiled food population, dark blue for the non-Roquefort cheese population, purple for the Roquefort cheese population and light blue for the Termignon cheese population. The results of the global test for a population effect is given at the top of each panel. Pairwise significant differences are indicated by asterisks. The boxplots represent the median (center line), the first quartile and third quartile (box bounds), the maximum and minimum excluding outlier points (whiskers), points being the outliers, i.e. with values either below the first quartile minus 1.5 fold the interquartile range or above the third quartile plus 1.5 fold the interquartile range. [file 43008_2024_167_MOESM9_ESM.png]
